# Unveiling the Role of Metal Ion Concentration versus Immune Sensitization in Orthodontic Patients—A Long-Term Prospective Evaluation

**DOI:** 10.3390/jcm13154545

**Published:** 2024-08-03

**Authors:** Nusha Paschaei, Wolf-Dieter Müller, Franziska Schmidt, Katrin Hüsker, Volker von Baehr, Nikolaos Pandis, Paul-Georg Jost-Brinkmann, Theodosia Bartzela

**Affiliations:** 1 Department of Orthodontics and Dentofacial Orthopedics, CharitéCenter for Oral Health Sciences CC 3, Charité—Universitätsmedizin Berlin, Corporate Member of Freie Universität Berlin and Humboldt-Universität zu Berlin, Aßmannshauser Str. 4–6, 14197 Berlin, Germany; 2 Department of Prosthodontics, Geriatric Dentistry and Craniomandibular Disorders, CharitéCenter for Oral Health Sciences CC 3, Dental Materials and Biomaterial Research, Charité—Universitätsmedizin Berlin, Corporate Member of Freie Universität Berlin and Humboldt-Universität zu Berlin, Aßmannshauser Str. 4–6, 14197 Berlin, Germany; wdmue54@gmail.com (W.-D.M.); franziska.schmidt2@charite.de (F.S.); 3 Department of Immunology, IMD Institute of Medical Diagnostics Berlin-Potsdam GbR, 12247 Berlin, Germany; k.huesker@imd-berlin.de (K.H.); v.vonbaehr@imd-berlin.de (V.v.B.); 4 Department of Orthodontics and Dentofacial Orthopedics, Dental School/Medical Faculty, University of Bern, Freiburgstraße 7, 3010 Bern, Switzerland; npandis@yahoo.com; 5 Department of Orthodontics, Faculty of Medicine Carl Gustav Carus, Technische Universität Dresden, Fetscherstr. 74, 01307 Dresden, Germany

**Keywords:** orthodontics, fixed orthodontic appliance, multibracket, immune system, corrosion, ion release, ICP-MS analysis, lymphocyte transformation test, in vivo

## Abstract

**Background:** This longitudinal prospective study aimed to assess orthodontic patients’ immune system response to metal ion release in saliva. **Methods:** Thirty adult patients (18–35 years) were equally divided into three groups: groups at the end (G1) and beginning (G2) of multibracket appliances (MBA) treatment and a non-treated control group (G3). Participants were evaluated at four timepoints within 21 days, with saliva samples being analyzed for metal ion concentrations and blood for the lymphocyte transformation test (LTT). **Results:** There were no significant differences between groups or timepoints for saliva. LTT analyses revealed hypersensitivity in one-third of all patients and 50% of G2 for nickel, with three developing sensitizations after MBA insertion. All nickel-sensitized patients exhibited varying elevated saliva nickel concentrations. The most nickel-sensitized patients had low ion saliva loads. In borderline nickel-sensitization cases, saliva ion concentrations were up to 20 times higher than the reference. Hypersensitivity to palladium, gold, and mercury was also observed. **Conclusions:** These findings indicate that increased MBA ion release was not inherently linked to the immune response (Type-IV sensitization), as reactions occurred even with ion levels below thresholds. This underlines the need for a comprehensive evaluation of the immune response to metal ion release in orthodontic patients.

## 1. Introduction

In orthodontics, traditional teeth alignment involves the use of fixed multibracket appliances (MBA), consisting primarily of bands, brackets, archwires, steel ligatures, and auxiliaries. These components are typically made from metal alloys, primarily stainless steel, nickel–titanium, or nickel–cobalt alloys [1].

While heavy metal contamination of drinking water is already a significant health issue that can lead to the disruption of metabolic processes [2], additional chronic exposure to orthodontics has also been investigated [3]. Metal ions released from orthodontic appliances [1,4,5] are a severe concern [6] as these ions are biologically available in a dissolved state and can affect the immune system [7].

Factors such as dietary habits [8], personal oral hygiene and oral microflora [9], the resulting pH and temperature fluctuations [10,11], and other modulating factors [9,12,13] can influence the corrosion process and thus, the metal ion release [14]. While most studies report metal ion concentrations below toxic levels [12,13,14] and consider dietary intake [9,13,15], there is a potential oversight of non-quantitative effects. Table 1 summarizes published studies and known outcomes focusing on corrosion, metal ion releases, and their impact on human health.

Therefore, there is an urgent need for clinical trials and protocols to monitor short- and long-term effects, as in vitro studies cannot fully reflect intraoral conditions [6,9,10,11,16].

It is established that ion release of metals such as Nickel (Ni) and Cobalt (Co) can induce DNA alterations in oral mucosa cells, leading to allergenic, cytotoxic, genotoxic, or mutagenic effects [1,11,17,18,19].

However, the focus should not solely revolve around toxic thresholds but also on whether non-toxic concentrations are sufficient to cause patients’ DNA alterations [18,20]. While a toxic effect typically requires larger amounts of a substance, allergic reactions [7] and hypersensitivity can occur with much smaller quantities [15], particularly in sensitized patients [21]. Nevertheless, higher metal exposure increases the risk for sensitization in cases of excessive surface wear or corrosion by the innate immune system’s recognition of these ions as danger signals [22,23]. The reference values are set as limits by the laboratories evaluating the data based on the general population norms. It is essential to know that in the case of an MBA, these limits can be consistently underestimated due to long-term exposure to low doses.

Even if low doses prove sufficient for allergy or sensitization assessments, developing a protocol to identify the causative factors locally in saliva through metal ion release when a positive LTT occurs during orthodontic treatment is worthwhile.

According to the systematic review and meta-analysis of Gölz et al., the prevalence of Ni hypersensitivity is 19%, with women showing a higher susceptibility [24]. Research has indicated immunological effects such as allergies, autoimmune diseases, or chronic inflammation associated with intraoral metal restorations [4,7]. Consequently, it is crucial to consider immunological reactions and potential long-term effects in orthodontic patients undergoing metal-based MBA treatment, which can span several years.

Therefore, with direct implications in clinical practice, this pilot study aimed to gain insight into the potential short- and long-term effects of metal ion release from MBA and detect immunological reactions that might occur independently of the quantity of metal ions in saliva. This study’s findings could significantly affect how we approach orthodontic treatment, material selection, and overall patient care.

The null hypothesis stated that

- the amount of metal ions released by fixed orthodontic appliances in saliva is not sufficient to cause toxic or allergic reactions; however, prolonged exposure could potentially lead to sensitization in these patients;
- positive sensitization shows a positive correlation to local exposure in saliva.

**Table 1:** Overview of in vivo studies conducted between 2001 and 2022 on metal ion release from orthodontic appliances and intraoral or immune system findings.

Author(s) (Year)	Observational Period (OP)	N (Age)	Dental Material and Immune System Tested	Metal Ions	Procedures Employed	Outcome (Metal Release, Immune System, Intraoral Findings)
Agaoglu et al. (2001) [25]	1 OP -unique timepoint for each group: before MBA insertion, 1st w, 1st m, 1st y, 2 y later	100 -5 groups (12–33 y)	-MBA: 4 bd, 20 br and wires: 1. NiTi; 2. SS -Saliva and blood	Ni, Cr	Electrothermal AAS	-Ni, Cr increase in 1st month -Ni, Cr decrease in 2nd year -no toxic levels -no relationship between Ni level in saliva and serum
Faccioni et al. (2003) [1]	1 sample collection	85: 55 MBA, 30 control (12–35 y)	Buccal mucosa cells	Ni, Co	-ICP-MS -Comet assay	-2.8-fold–3.4-fold higher ion levels -DNA damage
Fernandez-Minano et al. (2011) [17]	30 d -2 timepoints: -before -30 d after orthodontics	15 (12–16 y)	-SS, Ni, Ti, Ni-free -Buccal mucosa cells	Ti, Cr, Mn, Co, Ni, Mo, Fe	-ICP-MS -Comet assay	-Ni free MBA higher levels of Cr and Fe -Ti alloys induced increased levels of Mn -Ti alloys no toxic effect -SS and Ni-free MBA greater DNA damage
Nayak et al. (2015) [26]	Orthodontic treatment -pre-treatment -after aligning -10–12 m after treatment-beginning	30 (10–25 y)	-Br, bd and wires: 1. NiTi, 2. NiTi (heat activated), 3. SS -saliva (after 30 s rinsing)	Ni, Cr	-ICP-MS	-Ni and Cr increase after aligning phase -after 10–12 m, increased Cr and decreased Ni levels -concern about biocompatibility and allergic reaction frequency
Gölz et al. (2016) [27]	8 w: before treatment, after br and bd placement, before and after archwire insertion -4 and 8 w later	30 (10–13 y)	-br (self-ligating), bd -wires: NiTi -unstimulated saliva	Ni	-ICP-MS	-significant increase after br/bd insertion -decrease after 4 w -below dietary intake
Pazzini et al. (2016) [28]	-before treatment -12 m: every 3 m -1 m after removal	42 allergic patients (10–45 y)	-21 conventional br -21 nickel-free br	Ni	-patch test -gingival index -blood	-both groups: increased basophils -conventional group: decreased eosinophils and immunoglobin E -Ni levels: increased while treatment; decreased 1 m after -Ni-free br while treatment: gingival health, smaller blood changes
Quadras et al. (2019) [29]	1.5 y -5 timepoints: before archwire insertion; after 1 week, 3 m, 1 y, and 1.5 y	80 (15–40 y) -50 MBAs -30 controls	-20 br, 4–8 bd: SS -2 wires: NiTi/SS -saliva (after rinsing); blood	Ni, Cr, Zi	AAS	-Increase before and after insertion of the appliance -below toxic levels -after 1.5 y: significant difference between treated and control group
Lucarelli et al. (2020) [30]	1 y 2 tests: before and 1 y after treatment	60	-Ti rapid palatal expander and corrector	-Ni (allergy) -Ti	-patch test	-first test: sensitivity in 8 patients (2 males/6 females) -second test: 37 positive nickel sensitizations (25 females) -Ti appliances have high resistance and no allergic reaction
Zigante et al. (2022) [31]	6 w–1 y treatment	235 (11–45 y)	-orthodontic appliances -oral mucosa, gingiva, tongue, lips	-Ni -Ti	-Patch test -clinical signs	-clinical predictors of metal sensitization: adult age, female sex, exfoliative cheilitis, history of contact hypersensitivity to metals and piercings -patch test alone not conclusive for allergies

**N**—Number of patients, **s** second(s), **h**—hour(s), **d**—day(s), **w**—week(s), **m**—month(s), **y**—year(s), **bd**—band(s), **br**—bracket(s), **MBA**—multibracket appliance, **NiTi**—nickel–titanium, **SS**—stainless steel, **AAS**—atomic absorption spectrophotometer, **ICP-MS**—inductively coupled plasma-mass spectrometry.

## 2. Patients, Materials, and Methods

Ethical approval for the study was obtained from the Ethics Committee of the Charité—Universitätsmedizin Berlin, Germany (EA2/124/16; 21.03.2017). Additionally, the study was registered in the German registry for clinical trials (ID: DRKS00027231).

### 2.1. Participants’ General Characteristics and Inclusion and Exclusion Criteria

The study included thirty healthy individuals aged 18 to 35 divided into three groups. Group 1 (*n* = 10) comprised individuals in the debonding stage at the end of MBA treatment, while Group 2 (*n* = 10) comprised individuals in the bonding stage at the beginning of treatment. Group 3 (*n* = 10) was a non-treated control group.

Participants were recruited from the Charité—Universitätsmedizin Berlin, Department of Orthodontics, and two orthodontic practices in Berlin. Patients’ demographic and selection criteria are outlined in Table 2.

The MBA used in the study (3M, Dentaurum, Orthana, and Forestadent) comprised a minimum of ten metal brackets, two or more bands, two archwires, wire ligatures, and metal auxiliaries resulting in different alloys and compositions. All debonding group (G1) registrations were conducted without retention wires or metallic appliances. Only patients without additional intraoral metal restorations were included.

### 2.2. Material and Methods

Participants were informed and instructed about the study procedure and signed the informed consent forms. Each patient received a pseudonym and was evaluated anonymously. As shown in Table 3, all participants were examined at four study timepoints (T1–T4) over 21 days, with the first timepoint reflecting different baseline conditions for the study participants: T1 represents a timepoint without metal (MBA) for the bonding group (G2) and for the debonding group (G1), it is the only timepoint with MBA.

**Figure 1:**
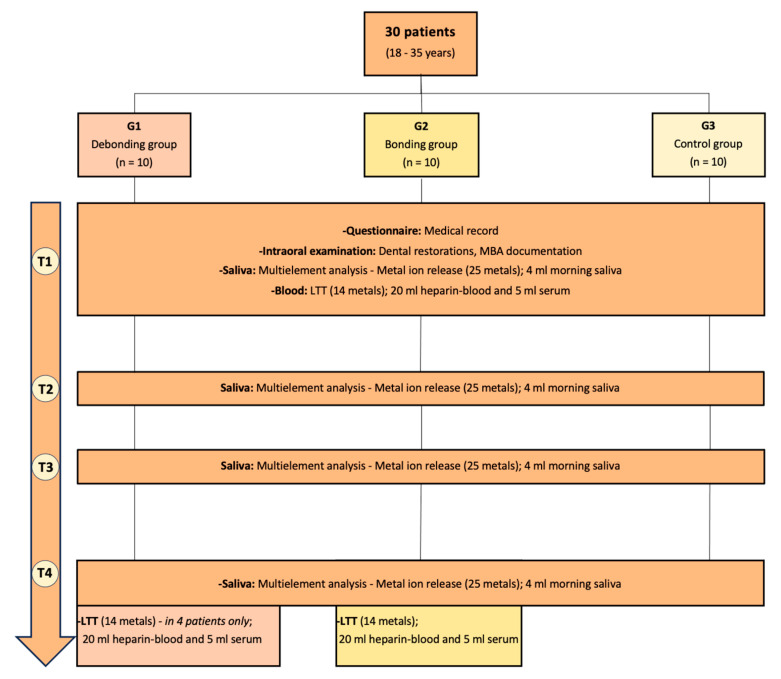
The flowchart illustrates the data collected at T1–T4 from the three groups of patients. **T1** day 0, **T2** day 1, **T3** day 7, and **T4** day 21. **LTT:** Lymphocyte transformation test (**T1:** G1, G2, G3; **T4:** G2 and four patients of G1).

To assess the metal ion release, 4 mL of non-stimulated saliva was collected in a saliva collection tube from each participant before breakfast and tooth brushing at every time interval (T1–T4) (Table 3 and Figure 1).

Blood samples were collected for the three groups at T1 and partially at T4, with 2 × 10 mL heparin blood and 10 mL for 5 mL of serum used for the LTT. Sensitization was tested at T1 using the LTT for all participants (Figure 1). In the bonding group (G2), sensitization through the MBA was tested after three weeks at timepoint T4. Additionally, in patients with reported additional clinical symptoms or abnormalities such as changes in the oral mucosa (irritation or inflammation) or frequent headaches, LTT analysis was conducted. Four patients exhibited these symptoms and LTT analysis was conducted at T4 (Figure 1). The examination of timepoints T1 and T4 was undertaken in the dental facility, while the patient at home performed T2 and T3 independently. The researcher (N.P.) collected the data and accessed the corresponding laboratory results after completing all registrations to ensure unbiased evaluation.

### 2.3. Analytical Methods

**Metal analyses in saliva:** The saliva samples underwent a multielement analysis (MEA). All specimens were stored at 4 °C and analyzed within 72 h of sampling. Saliva samples were diluted 1:20 in high purity 1% HNO3 (Suprapur, Supelco) or 1% HCl (Suprapur, Merck). The subsequent multielement analyses were performed using inductively coupled plasma mass spectrometry (ICP-MS, ICap Q, Thermo Fisher; Waltham, MA, USA) in the collision/reaction cell mode, with external and internal standard calibration (Elemental Scientific).

The salivary metal levels, quantified in μg/L, of the following 25 metals were determined: Au, Pd, Pt, Ga, In, Ir, Cu, Ag, Sn, Hg, Ce, Cr, Co, Mn, Mo, Ni, V, Al, Sb, Ba, Sr, Zn, Zr, Cd, and Ti. Based on the metals associated with sensitization, we limited our testing accordingly. Therefore, the following metals were tested: Ni, Au, Pd, and Hg.

**Lymphocyte transformation test:** The lymphocyte transformation test (LTT), an indicator for type IV allergic reaction sensitization, was performed on all participants, as shown in Figure 1. A total of 30 mL of blood was collected for each patient transported to the laboratory within 24 h. LTT was routinely evaluated at the baseline (T1) for all participants and at T4 for the bonding group (G2) following orthodontic treatment. Furthermore, it was also selectively conducted on the debonding group (G1) at T4 (Figure 1).

The LTT investigated possible cellular sensitization to 14 metals: Cr, Co, Pd, Ag, Al, Sn, Cu, Hg, Au, Ni, Cd, EtHg, Mo, and Pt, following the methodology described by von Baehr [32]. Stimulation indices (SI) were calculated for each allergen, derived from the mean value of three parallel tests per patient. This is formed as the quotient of the allergen-induced and the restimulated thymidine incorporation rate (blank value in cpm). An SI > 3 indicated the presence of allergen-specific T cells in the patient’s blood (positive result for cellular sensitization), while an SI < 2 was considered negative. Results between 2 and 3 were deemed borderline (weak or questionable sensitization). This scoring method aligns with von Baehr et al. [32] and the medical findings of the IMD laboratory (Berlin).

Negative and positive controls were performed in parallel for each test to detect non-specific cell reactivity and ensure that the lymphocytes had sufficient vitality and reactivity during testing. This excluded, for example, sample transport damage, the influence of medication, or handling errors in the laboratory.

### 2.4. Statistical Analysis

The reference values from the IMD laboratory served as a norm for deviations. Microsoft Excel was used to overview concentration values and implement descriptive statistics. First, tabulations per group (*n* = 10) across time for saliva and LTT for the selected metals were conducted as percent and counts. The ion concentrations were converted to binary (below/above threshold) and a generalized estimating equation (GEE) logistic regression was employed to analyze the group and time effects on ion concentrations for nickel, gold, palladium, and mercury.

The significance level was set at 5%. All statistical analyses and visualizations were performed using Microsoft Office, Stata 18 (Stata Corp, College Station, TX, USA), and the R programming language (Version 17; R Foundation for Statistical Computing, Vienna, Austria).

## 3. Results

A total of two experimental groups and one control were distinguished in the comparison. First, they were examined independently and then comparatively. In the morning saliva, the release of metal ions was measured in all groups at all examination timepoints (T1–T4). This allowed assessment of the course before, during, after, and without orthodontic treatment. The control group demonstrated the MBA-independent fluctuations. Our patients reacted positively or borderline to nickel (Ni), gold (Au), palladium (Pd), and mercury (Hg) and occasionally showed combination reactions. For this reason, these metals were evaluated for quantitative and comparison analyses in saliva as local spread. Correlations with concentration dependence were sought, where Ni especially appears to be the most important and representative of the results regarding MBA.

### 3.1. Metal Ion Concentration in Saliva

**Figure 2:**
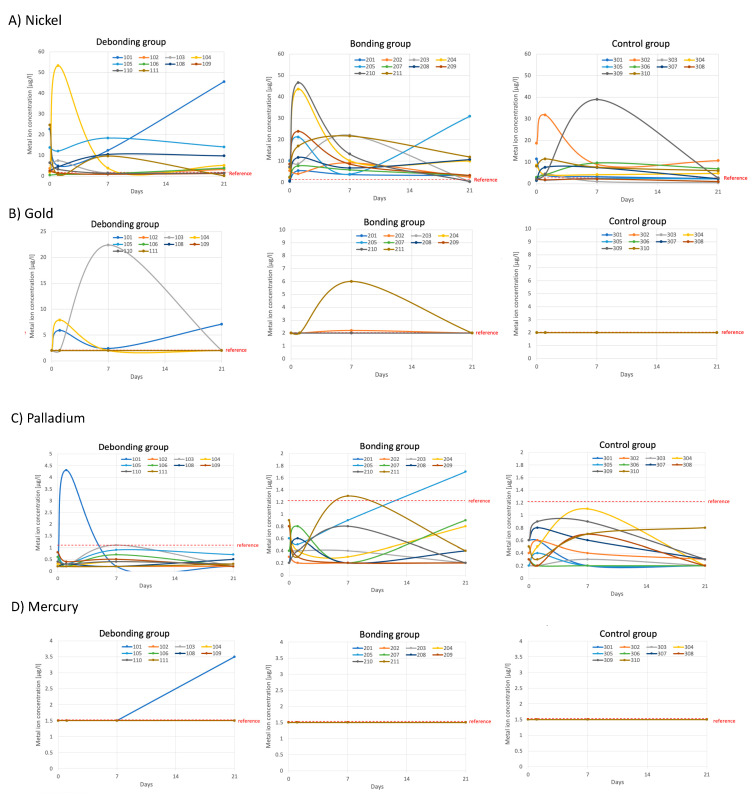
Time course (0–21 days) of metal ion concentration [µg/L] (**A**: Ni, **B**: Au, **C**: Pd, and **D**: Hg) in saliva in each group (debonding group, bonding group, and control group). **Red dotted line:** reference value from the IMD-laboratory; **101–111:** debonding group patients, **201–211:** bonding group patients, and **301–310:** control group.

**Figure 3:**
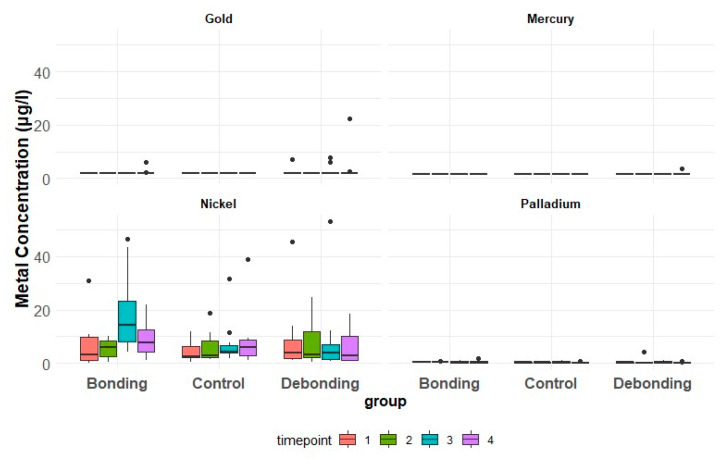
Box and whisker plots of metal ion concentrations [μg/L] of Ni, Au, Pd, and Hg in saliva for each group (*n* = 10) per timepoint.

Figure 2 provides individualized assessments of the patients’ metal concentrations within their respective groups. Although graphical representations showed fluctuations, no significant differences were found statistically for time and groups. Descriptive statistics for metal ion concentrations [µg/L] in saliva according to the group and timepoint are presented in Table 4 and Figure 3. A GEE logistic regression model for the effect of group and time was applied and the results are shown in Table 5. The odds of encountering a high Ni concentration in the control group were 0.47 times those in the debonding group (*p* = 0.34) and 0.57 times those in the bonding group (*p* = 0.49). The overall test for the group (*p* = 0.63) and time (*p* = 0.65) showed no significant differences. The odds of having a high concentration of gold were 1.89 times higher in the debonding than in the control group; there was, however, no statistically significance difference (*p* = 0.55). Group and time (*p* = 0.21) were not significant overall. Palladium showed a 0.48 times higher concentration in the debonding group than in the control (*p* = 0.54). Overall, the *p*-value for time was 1 and for the group, it was 0.54.

#### 3.1.1. Ni

**Debonding Group:** With only 14 exceptions of 120 measurements, nearly all patient values at each timepoint exceeded the reference value of <1.2 µg/L. The mean concentration decreased after treatment (T2) in six patients and increased in four patients. From T3 to T4, concentrations increased in seven patients, notably, in one patient showing a steady rise from T2 to T4, with T4 being twice as high as T1 (Figure 2A).

**Bonding Group:** Concentrations predominantly increased after bonding at T2, with variations among patients. Concentration values generally ranged from 0.5 to 46.6 µg/L (Figure 2 and Figure 3). Two patients displayed a more than twofold increase in metal concentrations after bonding (Figure 2A).

**Control Group:** Measured concentration values were mainly above the reference value, with only two exceptions. They followed a different course compared to the MBA treatment groups (Figure 2A). The concentration values fluctuated between 0.5 µg/L and 39 µg/L with a mean value across all of the timepoints of 6.5 µg/L. Notably, the two patients with the highest values (31.8 µg/L and 39 µg/L) remain below the maximum values observed in the G1 and G2.

#### 3.1.2. Au

**Debonding group:** In the majority of patients (*n* = 7), all measured values were consistently below the reference value of <2 µg/L (Figure 2B). Three patients showed increases in concentration after debonding, with this group showing the most significant Au concentration. Specific patient observations (Figure 2B) included the following. One patient displayed an increase in concentration of 195% at T2 compared to the initial and reference values, which subsequently decreased. Another patient showed an increase to 22.4 µg/L at T3, while the third patient demonstrated an increase to 7.9% at T2.

**Bonding group:** Following bonding in two patients, the concentrations increased at T3. Both showed a return to the reference value at T4 (Figure 2B).

**Control group:** Compared to both treatment groups (G1 and G2), it was remarkable that no patient showed increased salivary Au concentrations.

#### 3.1.3. Pd

**Debonding group:** All Pd concentrations, except in one patient (Figure 2C), were below the reference value of <1.2 µg/L. The largest fluctuations consistently occurred at T3 in most patients, with a noticeable pattern of returning to approximate baseline values at T4.

**Bonding group:** Most values were below the reference value, with considerable variability. The range extended from a minimum of 0.2 to a maximum of 1.7 µg/L (Table 4). Additionally, we have noted specific patterns in individual patients, such as one showing a steady increase from T2 to T4 and another displaying an increase at T3 (Figure 2C).

**Control group:** Values varied between 0.2 and 1.1 µg/L, with all being below the reference value.

#### 3.1.4. Hg

Only one patient (Figure 2D; 101) from the debonding group exceeded the reference level of 1.5 µg/L with 3.5 µg/L at one timepoint (T4). For this reason, no inferential statistics were possible.

### 3.2. Lymphocyte Transformation Test

There were differences in sensitization in the groups, timepoints, and expression strength (stimulation indices = SI). Detailed information comparing T1, T4, and gender distribution is shown in Table 6.

#### 3.2.1. Debonding Group (G1)

In G1, no immune changes were originally anticipated after MBA removal. However, the LTT was repeated at T4 in four exceptional cases reporting clinical abnormalities. Overall, 3 out of 10 patients showed (borderline) sensitization. Specifically, one patient (101) initially exhibited no sensitization at time T1 but positive sensitization at T4 to Pd (SI = 3.3) and borderline sensitization to Ni (SI = 2.8). One patient (102) displayed borderline sensitization (SI = 2.4) to Hg at T1 and none at T4. Conversely, the last patient, who was tested twice (111), demonstrated borderline sensitization (SI = 2.5) to Ni at T1, whereas testing at T4 revealed no sensitization.

#### 3.2.2. Bonding Group (G2)

Overall, of the 10 bonding patients studied at two timepoints, 50% showed positive sensitization (*n* = 5) and all were more severe than in the debonding group. Two patients (202 and 204) showed a positive Ni sensitization before MBA at T1 (SI = 4 and SI = 4.4, respectively). Three weeks later, at T4, following MBA treatment, both patients demonstrated a negative result in LTT (SI < 2). Before MBA therapy at T1, patients 207, 208, and 211 displayed negative LTT results and three weeks after bonding (T4), they reacted positively, indicating a new sensitization. Patient 207 exhibited an SI of 17.5 for nickel; patient 208 of 4.8; and patient 211 had an SI of 4.1. At T4, patient 211 displayed a borderline sensitization, with an SI of 2.3 for Pd.

#### 3.2.3. Control Group (G3)

Sensitization testing was conducted only once, at T1, without MBA treatment. Three patients (303, 305, and 307) tested positive for Ni sensitization. Patients 305 and 307 exhibited an SI of 3.3, while patient 303 had a notably higher SI of 46.3 for Ni and a borderline sensitization for Au with an SI of 2.9. Patient 307 also had an additional borderline sensitization to Hg. Furthermore, patient 310 demonstrated sensitization solely to Hg with an SI of 3.4.

To summarize, across all three groups, 10 patients tested positive regardless of time and metal. Two others showed borderline sensitization. In total, five female and five male patients showed borderline or positive Ni-sensitization, one female and one male patient showed borderline or positive Pd-sensitization, one female positive and two male patients showed borderline sensitizations to Hg, and just one female patient a borderline LTT to Au (Table 6). Cross allergies were detectable in two patients. They showed combined sensitization to Ni and Pd, one to Ni and Au, and one to Ni and Hg.

### 3.3. Correlation between LTT and Metal Ion Concentrations

#### 3.3.1. Ni

**Debonding group:** Patient 101 showed a negative immune reaction at T1, followed by a borderline LTT at T4, with Ni concentrations above the reference value. Conversely, in patient 111 of the same group, a borderline response was noted at T1 and a negative reaction at T4, with a concentration below the reference value (Table 6; Figure 2A).

**Bonding group:** Five patients showed a positive Ni sensitization. Patients 202 and 204 exhibited positive LTTs at T1 with an SI of 4 and 4.4 (Table 6). Despite vastly different saliva metal ion concentrations between these two patients (Figure 2A, 202 vs. 204), both showed relatively low concentrations above the reference value during the positive reaction at T1 (without MBA). Concentrations increased at T3 (Figure 2A) and T4 and both patients tested negative. The remaining three patients in the bonding group (207, 208, and 211) showed positive LTT at T4. Salivary Ni concentrations increased from T1 to T4 in all patients, surpassing the reference value (Figure 2A); but, in patient 207, it remained lower and closer to the reference at T4 (Figure 2A, 207).

**Control group:** The mean values of G2 (T1, without MBA) and G3 were comparable (Table 4). The most remarkable patient (303) with an SI of 46.3 (Table 6, G3) showed one of the lowest saliva concentrations fluctuating around the reference value (Figure 2A). She had long-standing reddish skin symptoms, primarily in the neck and chest region, but had not undergone prior allergy testing. Despite minimal nickel exposure and no increase in metal concentrations in saliva, symptoms persisted, highlighting the complexity of clinical correlations. The patient had no history of piercings or dental restorative treatment. Following testing, dietary consultation was provided by the laboratory physician, revealing that the patient’s mother had a significant preexisting allergy history.

#### 3.3.2. Au

The only borderline sensitization occurred in the control group (Table 6) but no increases in saliva concentration were detected (Figure 2A).

#### 3.3.3. Pd

**Debonding group:** One patient (101) exhibited positive LTT (SI 3.3) at T4 and showed solely an increased Pd concentration above the reference in this group at T2.

**Bonding group:** Similar patterns were observed in the borderline sensitization at T4 (Table 6, G2, patient 211). Patient 211 showed an elevated Pd concentration above the reference value at T3 (Figure 2C) and a dual sensitization to Ni.

**Control group:** Fluctuations were noticeable but there was no LTT reaction.

#### 3.3.4. Hg

Only a singular saliva concentration elevation was identified, although this patient did not exhibit positive sensitization. The borderline-sensitized patient in the debonding group (Table 6) did not exceed the reference value.

## 4. Discussion

MBA is commonly used in orthodontics, releasing metal ions affected by various factors. Even though studies suggest low ion levels, clinical trials are necessary to evaluate long-term effects. Metals like Ni and Pd have been linked to oral lesions and immune responses [33]. The biocompatibility of orthodontic materials represented a worrying topic [30]. Studies have shown that Ni concentrations increase after bracket and band insertion in MBA patients [27]. Additionally, during the aligning phase [26], saliva exhibited higher concentrations of Ni and Cr, which are considered mutagenic, cytotoxic, and allergenic [8,34]. Elevated DNA damage induced by Ni and Co in buccal mucosa cells has also been reported [1]. Elevated basophil levels [28] and various reactions, such as gingivitis, which can occur eight months after a trans palatal arch, have also been linked to Ni allergies [35]. Nevertheless, concentrations below toxic limits [25] and complementary results from reviews in which there is no increased prevalence associated with Ni allergies from orthodontic treatments for Ni allergies [21] have been reported.

Therefore, the tested hypothesis in this study suggests that the amount of metal ions released by MBA was not sufficient to cause acute toxic or allergic reactions but could potentially induce sensitization in prolonged metal ion exposure. It was assumed that in cases of positive sensitization, there would be a positive correlation with local exposure to saliva. This assumption is based on previous findings that showed a correlation between immune response and metal levels in serum caused by joint implants [36].

Elevated levels of metal concentrations were detected in saliva, particularly in the MBA groups (G1 and G2) and post-MBA treatment (T2–T3), showing the most notable variations. The fluctuations observed in the control group were lower than in the treatment groups; however, they demonstrated dependencies on the individual lifestyle and highlighted their relevance compared to reference values. The MBA treatment did not exhibit any immediate acute or toxic effects, as expected. However, it did induce sensitization, which was detectable through LTT, underscoring its impact. Primarily due to borderline reactions and a lack of association with MBA, Au, Pd, and Hg are considered questionably in the interpretation. While nickel allergic contact dermatitis is the most prevalent delayed-type hypersensitivity reaction affecting the skin globally [37], Ni also demonstrates the most significant findings in this study. We can conclude that even low concentrations of sensitizing agents can trigger symptoms in sensitized patients despite the absence of clinical correlations in all patients. This is also reflected in recent studies, which showed that immunological responses can be observed even at low doses upon re-exposure [38]. Patients’ experiences vary widely, with some exhibiting symptoms such as headaches, altered taste, and skin changes following positive LTT results, while others remain asymptomatic, making preventive detection challenging. Increased metal ion exposure and the occurrence of the sensitization reaction (SI) could not be confirmed by the results. Sensitization was demonstrated without the anticipated exposure ion levels. The metal ion levels showed a statistically non-significant increase after bonding but did not show the expected decrease after debonding during the observation period.

In a study of patients with connective tissue disease, Ni hypersensitivity was reported [5]. In our research, the most frequent allergens besides Ni were Au, Hg, and Pd. Chronic exposure to low levels of metals can cause sensitization to Ni, Hg, and other metals [21]. This phenomenon could be affirmed in the treatment groups at T4. However, it is not possible to draw any definitive conclusions about the MBA regarding Au and Pd without examining the chemical composition. The reference values provided by the laboratory, representing the average population, have been surpassed in most cases. Statistical analysis was challenging due to the small patient sample.

### 4.1. Ni

The current study revealed a consistent increase in the saliva Ni concentration across all groups and timepoints, although the patterns varied (Figure 2A), and positive sensitizations were registered. Treatment groups showed higher metal ion concentrations compared to the control. More fluctuations were observed after the MBA treatments (T2–T3), supporting that the release occurred mainly in the first phase of treatment [8]. Sensitizations often exhibit increased Ni concentrations in the treatment groups; however, not every elevated concentration can be associated with sensitization.

#### 4.1.1. Debonding Group

Definitive conclusions regarding concentration levels cannot be drawn due to the initially low concentration and borderline reactions. This suggests that the extent of metal ion release and potential for reactions may have been underestimated after debonding, as an initial decrease was anticipated.

#### 4.1.2. Bonding Group

Confirmation of the sensitization by MBA or activation of a latent sensitization was evident in patients 207, 208, and 211 (Table 6), wherein a previously negative LTT (T1) became positive at T4 (three weeks post-insertion). A positive LTT was also observed at low concentrations compared to the others, as seen in patient 207 (Figure 2A). The only group with all patient values consistently surpassing the reference value at T2 and T3 was G2. This implies a pronounced influence of metal insertion and localized after-effects, persisting firmly until the 7th day and that is still measurable after 21 days. Both positive reactions at T1 exhibited a similar SI of 4 and 4.4 (Table 6, G2) despite differing low metal concentrations (Figure 2A). Following an increase up to T3, a negative LTT at T4 was observed, potentially suppressing the positive sensitization.

#### 4.1.3. Control Group

The most substantial concentration independent confirmation is shown by the most conspicuous patient (303), with the highest SI and one of the lowest saliva concentrations. In general, the concentration values are above the reference value, and it can be discussed whether the reference could be defined as too low, which can only be compared with a larger and longer observation, or all patients receive too much exposure due to their average lifestyle. This finding supports the co-dependence on other factors already mentioned in the introduction [8,9,13]. The fact that the control group concentrations were below those of the treatment groups proves treatment dependence.

### 4.2. Au

The dependency analysis between quantity and sensitization was striking for gold. The only borderline sensitization was detected in the control group but there were no increases in saliva compared to the treatment groups. In summary, no connection could be determined.

### 4.3. Pd

Alterations exceeding the reference value and LTT (cross-) reactions were found solely within the treatment groups (Table 6). An association between the quantity of concentration (Figure 2C) and a time-delayed LTT (cross-) reaction could be assumed for Pd-induced sensitization. However, the connection to the MBA was also questionable.

### 4.4. Hg

No patient with borderline or positive LTT showed elevated levels at any time and there were no (time-delayed) correlations between LTT and saliva (Figure 2D; Table 6). The probability of sensitization did not appear to be linked to the quantity.

### 4.5. Metal Ions

There are various sources of heavy metal pollution, such as environmental contamination and agricultural practices like pesticides accumulating in the food chain and further in animals and humans [39]. The issue of heavy metal contamination in drinking water is also well-known [2]. Residues may remain in saliva after consumption of contaminated food and drinks. Apart from the potential MBA-independence of Au, Pd, and Hg, the presence of Au in G1 and G2 occurred unexpectedly, since precious metal alloys were not used here during orthodontic treatment. The patients did not wear any (gold) jewelry, appropriate cutlery was not provided, and no instruments with coatings were used that could explain the elevated Au concentrations.

Due to the potential synergistic and toxic effects of the combination of nonspecific metals [17], statements about toxicity based on separate individual evaluations may not be meaningful. Therefore, our hypothesis is that the absence of toxic effects can only apply to isolated instances if the values remain below the established toxic limits. According to Schmalz and Arenholt-Bindslev [21], a crucial aspect of all measurements is that the applied dose may not correspond to the effective dose.

### 4.6. Sensitization

Although the German Society for Dermatology recommends using the patch test to detect type IV metal allergies, the lymphocyte transformation test (LTT) was chosen for this study and was validated as an in vitro method for testing delayed hypersensitivity [40]. The LTT is particularly suitable for metals like nickel [39] and does not carry the risk of sensitizing a patient [40]. In contrast, skin testing can pose a risk of sensitization during application [41], which could explain sensitization in patients with a history of previous metal testing.

The assumption that oral exposure to Ni cannot lead to sensitization and that there would be no discernible increase in the rate of sensitization during orthodontic treatment [41] has been refuted (Table 6, G2, T4). Ni, which was tested as the most allergenic substance, has been proven to induce sensitization [29]. The null hypothesis was that the metal ion concentrations were sufficient to cause sensitization in the bonding group after MBA placement even when no toxic effects were shown. This hypothesis was confirmed in 3 of 10 G2 patients (30%) who had a negative LTT at T1 and showed positive sensitization to Ni at T4. Surprisingly, two other patients showed a positive LTT before MBA (T1) to Ni and a negative test on T4; which represents a similar result to G1, with a negative reaction and suppression of the immune response at the time of stress with metal release (for G1 = T1 and for G2 = T4) and during relief without MBA (G1 = T4, G2 = T1) positive sensitization. This could be due to the induction and proliferation of Ni-specific tolerance-inducing cells through continuous exposure to Ni following MBA insertion. Based on current knowledge, it is understood that Ni-specific T cells in the blood is normal but CD25(+) Treg cells in non-nickel-allergic individuals suppress the activation of nickel-specific T effector cells [42]. Overall, 50% of patients in this group had positive evidence of sensitization to Ni and one patient had borderline sensitivity to Pd. Cross-allergies between Ni and Pd, which seemed to be underestimated [33], were detected in two patients (Table 6).

In the case of borderline reactions, the exact significance should not be attributed to their interpretation. In the instance of a borderline reaction in G1 (T1) followed by a subsequent negative reaction, the result may be considered questionable or, potentially, the corresponding allergen was no longer a chronic burden in the body, causing the T-lymphocytes to disappear in the cells or decline in number in the lymph nodes so that they no longer circulate in the blood. If symptoms are suppressed by the body, the continuous struggle may have an impact and autoimmune diseases cannot be ruled out.

As highlighted by Ahlström et al., clinicians must acknowledge the potential for chronicity in hypersensitivity reactions. Typical clinical manifestations, such as allergic nickel dermatitis, occur with direct contact and predominantly affect the skin [43]. In patients with fixed orthodontic appliances, there is a higher incidence of (peri-)oral manifestations of hypersensitivity reactions [44]. But often, visible reactions are more likely to occur at a distance than at the local point of contact [20]. A study conducted in 68 orthodontic practices in the state of Hesse recorded more extraoral skin reactions than intraoral ones [43]. This observation aligns with the findings from our control group. Another hypothesis proposed that previous orthodontic treatment could cause immune tolerance. A similar tolerance hypothesis was already described in 1984 by Vreeburg et al., suggesting that the oral administration of nickel and chromium does not trigger hypersensitivity; it may even suppress it [44]. Fors et al. also suggested that using orthodontic appliances containing Ni before becoming sensitive to this metal (e.g., before getting an ear piercing) might decrease the occurrence of nickel hypersensitivity [45]. While no statistical significance was reported, there was a trend supporting that MBA treatment before ear piercing might have a protective effect against Ni sensitization. However, evidence suggests that ear piercing is not a critical factor for Ni contact allergy [6]. Although we did not specifically investigate the connection to previous piercings, it is worth noting that two sensitized patients in our study did not wear any jewelry, yet still showed sensitization. It must also be mentioned that after sensitization and primary contact, further treatment and metal replacement would already constitute a trigger.

Our study did not reveal any patients with positive sensitization to Cr or Co despite testing for these metals. Nevertheless, they should not be neglected as additional markers for genotoxicity [1].

Normalization through debonding was expected, as previously, an improvement after the removal of sensitizing metals was shown by Stejskal [4]. Regarding the influence on concentrations, we experienced fluctuations like increases in metal concentration after debonding. Withal, it must be emphasized that we also observed an improvement in the symptoms of patient 303 (Table 6, G3), with the highest SI through inquiry after partially adjusting her diet based on recommendations from the laboratory. Thus, even a reduction in exposure can lead to improvement and a decrease in constant stress and activation.

### 4.7. Patients, Materials, and Methods

Using different orthodontic materials, observation periods, and preconditions hardly allows for comparisons; particulary when different analytical methods are employed [9,29]. Additionally, factors such as the sample size (7–55 patients), number of groups, timepoints, and presence/absence of a control group must be considered [8]. Various in vivo studies investigated saliva, serum, oral mucosa cells, or urine [6,16]. The samples were often snapshots and did not reflect the load during the entire course of treatment [45]. While previous studies examined periods between 1 day and 2 months [6], we decided on a study duration of three weeks based on prior research indicating notable concentration fluctuations and the most substantial releases in the early post-treatment period, particularly within the initial seven days [13]. These concentrations subsequently returned to baseline levels by the end of the second week following bonding [27]. The first samples utilized in the present study were fasting unstimulated morning saliva, chosen primarily to investigate traces of corrosion. This saliva type should be collected undiluted immediately after getting up following reduced saliva flow at night. This timing is crucial for capturing any changes in saliva composition that may occur overnight due to decreased saliva production during sleep. Previous studies have mentioned variations in sample collection protocols, such as considering previous food intake or rinsing up to approximately 30 min before sampling [27] or employing a two-stage procedure before and after rinsing with distilled water [45]. It is essential for patients to correctly execute and conscientiously adhere to these protocols, as external influences like tooth brushing can falsify the results.

In the present study, patients aged 18 to 35 years were examined. Notably, orthodontic treatments mainly involve younger patients, and they likely have had less prior exposure. However, according to Janson et al. [41], no correlation appears to occur between age and allergic reactions. On the other hand, according to Zigante et al., adults face an elevated risk of developing allergic sensitization [46]. Results could therefore be higher in our (adult) study group. It is possible that Ni exposure promotes tolerance in children with MBA, which is no longer the case in adults. Therefore, future studies should include younger patients. According to the literature, women are more often affected [24,46]. While in our bonding group, more men were affected, in the control and debonding groups, more women were affected. However, this difference in our study may be attributed to the small sample sizes.

Referring to our study, it is important to note that while mean values or medians can be recorded for illustrative purposes, no conclusions can be drawn about individual patients. In this study, the immune response to MBA was not a matter of quantity of metal ions. Decisive statements regarding individual orthodontic material selection can only be made through individual considerations. Since it is not the single dose that causes various symptoms, a comparison with food intake at a certain timepoint [13] has no clinical relevance since it must be considered that exposure to orthodontic appliances takes place chronically over several years [7]. The effects of chronic exposure to metals in sensitive patients have already been shown in case studies for dental alloys and orthopedic implants [4], assuming that this also applies to long-term MBA treatments. Not reaching increased or toxic levels does not mean that the metals cannot destroy cells [1,17]. In addition to cellular effects, even very small (non-toxic) amounts of nickel release can cause allergic reactions in sensitive patients [47].

Consequently, relying only on concentration measurements is not an adequate approach to predict which patient might react sensitively. If allergies are suspected, it is advisable to conduct preventive LTT examinations, which can indicate possible treatment alterations after confirmation. If allergies are suspected after MBA placement, diagnostic testing should always be conducted in combination with other tests to assess both oral and systemic exposures accurately. However, the LTT may be suppressed (yielding negative results) during treatment (Table 6; G1, G2).

When discussing limitations and health consequences, sensitized patients should be excluded and managed differently. A suggested treatment protocol involves testing and avoiding exposure to sensitizing substances, mirroring standard practices in allergology and dermatology. Clinical signs can often be misinterpreted. Tests should also be considered for possible cross-allergies to inform patients about possible consequences for future or existing dental restorations and possible effects from combinations.

As previously mentioned, numerous factors, such as oral hygiene, pH value, diet, temperature, and others modulate the corrosion rate and thus the release of metal ions. Recent studies have even shown an increased release of metal ions following bleaching therapies [48]. For this reason, it is not feasible to reliably assess the release of various alloys in vivo without accounting for clinical factors. Consequently, additional studies are imperative to explore lifestyle and corrosion levels to draw plausible conclusions. Further assessments involving larger sample sizes and extended observation periods are indispensable to ascertain patient tolerance and establish selection criteria for orthodontic materials, particularly in individuals with underlying health conditions. The implementation of a prevention concept is recommended.

To the best of our knowledge, our pilot study represents the first comprehensive effort to examine patients at such a level on saliva metal ion measurements and to test for sensitization in three study groups and time in detail.

## 5. Strengths and Limitations of the Study

One strength of the study is the inclusion of three representative groups, allowing for a comprehensive evaluation of the effects of MBA in orthodontics, mainly through comparison with the control group and reference values. This approach increases the generalizability of the findings and enhances the study’s external validity. Additionally, the study focused on four timepoints and multiple biomarkers, including saliva and blood, providing insight into the dynamics of metal ion release following MBA treatment and sensitization and allowing for a comprehensive assessment. This multi-modal approach strengthens the study’s methodology and increases the reliability of the findings.

Due to the nature of the study, we were unable to blind or randomize the patients into groups. Ensuring consistency in sample collection is crucial for comparability. Samples collected independently by patients at home must be standardized and special strategies should be implemented to enhance compliance. Another limitation is the partial execution of the second LTT (T4) in the debonding group. Conducting tests twice for all patients could have offered more data for comparison. Yet, partial testing revealed an unexpected finding: sensitization changes post-MBA removal.

Furthermore, a limitation of the study was the sample size, which was constrained by the ethics committee’s age limits and our implemented exclusion criteria to improve comparability and minimize confounding results. The data were collected during the pandemic where the limited physical access to participants made the process more challenging. Future research should extend the duration and increase the sample size, including a wider age range, to strengthen the validity and enhance the generalizability of the finding and to capture long-term effects or changes in sensitization. A subgroup of analyses could explore potential confounding factors or interactions that may influence results, such as an increase in frequency in testing for capturing more longitudinal data to better understand the dynamics of sensitization.

Overall, the results show no clear relationship between ion release level and sensitization. This finding contributes to the existing literature and underscores the importance of further research.

## 6. Conclusions

Oral exposure to MBA can induce dose-independent sensitization, detectable as early as three weeks after insertion. Predicting the sensitization likelihood or extent based on saliva metal ion levels was not feasible. Although MBA treatment increased nickel concentration, no direct correlation was found between the concentration level and the likelihood or severity of sensitization, suggesting individual variability in response to metal exposure. However, in cases of confirmed sensitization, concentration measurements can pinpoint the exposure sources.

Even low concentrations in sensitized patients can trigger symptoms but not all patients may exhibit clinical signs. Given that 10 out of 30 patients exhibited positive sensitization, it is crucial to conduct patients’ thorough assessments for metal sensitization, implement mitigation strategies based on patients’ profiles to customize orthodontic treatment, and ensure careful selection of materials for patients with metal sensitization. Therefore, it is imperative to tailor orthodontic approaches for these patients to ensure optimal care and outcomes in clinical practice.

## Figures and Tables

**Table 2:** Patient general demographic and clinical characteristics.

	G1 (N = 10)	G2 (N = 10)	G3 (N = 10)
**Male/female (N)**	6/4	5/5	5/5
**Mean age (years)**	20.6	20.9	22.8
**Inclusion criteria**	- Removal of metal MBA in both jaws - No metal retention	- Insertion of metal MBA in both jaws	No history of orthodontic treatment or metal dental restorations
**Exclusion criteria**	- Immune system disorders - Metal allergy - Intraoral metal restorations - Smoking habit - Bruxism - Intake of supplements (vitamins/minerals) during study evaluation		

**N:** Number of patients; **G1:** Debonding group, **G2:** Bonding group, **G3:** Control group.

**Table 3:** Overview of the study groups and the registration timepoints.

	G1	G2	G3
**T1**	1st day; before MBA removal	1st day; before MBA placement	1st day
**T2**	1st morning after MBA removal	1st morning after MBA placement	2nd morning
**T3**	7th morning after MBA removal	7th morning with MBA	7th morning
**T4**	21st day after MBA removal	21st day with MBA	21st day

**G1:** Debonding group, **G2:** Bonding group, **G3:** Control group.

**Table 4:** Metal ion concentration levels in saliva (MEA [µg/L]) according to group and timepoint.

			Debonding			Bonding			Control		
	Time-Point	R [µg/L]	Median	First Q	Third Q	Median	First Q	Third Q	Median	First Q	Third Q
**Nickel [µg/L]**	T1	1.2	3.10	2.20	11.98	6.15	2.38	8.25	2.90	2.10	8.48
	T2	1.2	3.95	1.50	6.88	14.35	8.03	23.20	4.25	4.05	6.85
	T3	1.2	2.75	1.25	10.30	7.70	4.40	12.53	5.85	2.70	8.68
	T4	1.2	3.90	1.93	8.65	6.25	3.23	10.53	2.50	2.23	5.70
**Gold [µg/L]**	T1	2	2.00	2.00	2.00	2.00	2.00	2.00	2.00	2.00	2.00
	T2	2	2.00	2.00	2.00	2.00	2.00	2.00	2.00	2.00	2.00
	T3	2	2.00	2.00	2.00	2.00	2.00	2.00	2.00	2.00	2.00
	T4	2	2.00	2.00	2.00	2.00	2.00	2.00	2.00	2.00	2.00
**Palladium [µg/L]**	T1	1.2	0.35	0.23	0.55	0.40	0.30	0.75	0.35	0.23	0.58
	T2	1.2	0.25	0.20	0.30	0.40	0.30	0.48	0.45	0.23	0.60
	T3	1.2	0.40	0.20	0.65	0.25	0.20	0.70	0.50	0.23	0.70
	T4	1.2	0.25	0.20	0.30	0.30	0.20	0.70	0.20	0.20	0.30
**Mercury [µg/L]**	T1	1.5	1.50	1.50	1.50	1.50	1.50	1.50	1.50	1.50	1.50
	T2	1.5	1.50	1.50	1.50	1.50	1.50	1.50	1.50	1.50	1.50
	T3	1.5	1.50	1.50	1.50	1.50	1.50	1.50	1.50	1.50	1.50
	T4	1.5	1.50	1.50	1.50	1.50	1.50	1.50	1.50	1.50	1.50

**Timepoints:** T1 (Day 0), T2 (Day 1), T3 (Day 7), and T4 (Day 21); **R:** Reference value (laboratory), **Q:** Quartile; **Reference value R** provided by the laboratory.

**Table 5:** Generalized estimating equations’ (GEE) logistic regression results for the group and time effects on metal ion concentrations.

Metal			Odds Ratio (95% Confidence Interval)	*p*-Value
**Nickel**	**Group**	Debonding	0.47 (0.10, 2.23)	0.34
		Bonding	0.57 (0.12, 2.82)	0.49
		Control	Reference	
	**Time**	1	Reference	
		2	1.56 (0.25, 9.73)	0.63
		3	0.72 (0.15, 3.44)	0.68
		4	0.55 (0.12, 2.49)	0.44
**Gold**	**Group**	Debonding	1.89 (0.23, 15.37)	0.55
		Bonding	Not estimable	
		Control	Reference	
	**Time**	1	Reference	
		2	2.12 (0.30, 14.75)	0.45
		3	4.81 (0.76, 30.70)	0.10
		4	Not estimable	
**Palladium**	**Group**	Debonding	0.48 (0.05, 4.92)	0.54
		Bonding	Not estimable	
		Control	Reference	
	**Time**	1	Reference	
		2	1 (0.05, 18.67)	1
		3	1 (0.05, 18.67)	1
		4	Not estimable	

**Table 6:** Individual patients of the three study groups with at least one borderline or positive LTT test result (at timepoints T1 and T4).

		Timepoint T1 (Day 0)				Timepoint T4 (Day 21)			
Group	Patient (sex)	Pd	Hg	Au	Ni	Pd	Hg	Au	Ni
**G1**	101 (f)	1.0	1.0	1.0	1.0	3.3	1.4	1.4	2.8
	102 (m)	1.1	2.4	1.0	1.2	1.1	1.0	1.0	1.0
	111 (f)	1.4	1.8	1.7	2.5	1.2	1.0	1.0	1.0
**G2**	202 (m)	1.3	1.6	1.8	4	1.1	1.0	1.2	1.5
	204 (m)	1.7	1.4	1.4	4.4	1.1	1.3	1.5	1.4
	207 (m)	1.0	1.0	1.0	1.1	1.0	1.0	1.0	17.5
	208 (f)	1.3	1.2	1.5	1.6	1.0	1.0	1.0	4.8
	211 (m)	1.0	1.5	1.5	1.9	2.3	1.0	1.0	4.1
**G3**	303 (f)	1.0	1.0	2.9	46.3	-	-	-	-
	305 (m)	1.4	1.7	1.4	3.3	-	-	-	-
	307 (f)	1.2	2.6	1.7	3.3	-	-	-	-
	310 (f)	1.2	3.4	1.1	1.5	-	-	-	-

**Group G1** Debonding group, **G2** Bonding group, **G3** Control group; **Sex** = **f** (female), **m** (male); **Pd** Palladium, **Hg** Mercury, **Au** Gold, **Ni** Nickel; **[SI]** = stimulation index for the respective allergen; **>3 =** Safe existence of circulating allergen-specific T-cells in the patient’s blood (positive result = cellular sensitization; red color); **Values 2–3** are borderline (weak or questionable sensitization; apricot color), which should be controlled; **<2 =** Safe negative result (colorless).

## Data Availability

The data presented in this study are available on reasonable request from the corresponding author and partly at DRKS (ID: DRKS00027231).
